# Folate in the United States Population and its Association with Congestive Heart Failure

**DOI:** 10.31083/j.rcm2502039

**Published:** 2024-01-29

**Authors:** Longbo Wang, Fangcong Yu, Jiaran Shi, Tianxin Ye, Yunping Zhou, Zhuonan Sun, Jinxiu Yang, Xingxiang Wang

**Affiliations:** ^1^Department of Cardiology, The First Affiliated Hospital of Zhejiang University School of Medicine, 310003 Hangzhou, Zhejiang, China

**Keywords:** congestive heart failure, red blood cell folate, United States, dietary folate equivalents, restrictive cubic spline

## Abstract

**Background:**

To investigate the relationship between red blood cell (RBC) folate and congestive heart failure (CHF).

**Methods:**

We extracted the concentrations of RBC folate and collated CHF information from the National Health and Nutrition Examination Survey (NHANES) survey (12820 individuals). Weighted univariate logistic regression, weighted multivariate logistic regression, and restrictive cubic spline (RCS) were used to assess the relationship between RBC folate concentrations and CHF.

**Results:**

The unadjusted model showed that the highest tertile group of RBC folate concentration was significantly associated with a higher risk of CHF compared to the lowest tertile group of RBC folate levels (odds ratio [OR] = 3.09; 95% confidence interval [CI], 2.14–4.46). Similar trends were seen in the multivariate-adjusted analysis (OR = 1.98; 95% CI: 1.27–3.09). The OR was >1.0 when the predicted RBC folate exceeded 2757 nmol/L in the RCS model, indicating that the risk of CHF was low and relatively stable up to a predicted RBC folate level of 2757 nmol/L, but began to increase rapidly thereafter (*p* = 0.001).

**Conclusions:**

The risk of CHF may be increased either by high RBC folate concentrations (highest tertile of RBC folate or >2637 nmol/L) or by folate deficiency. Considering the two sides of the association between RBC folate and CHF, there is a need for large-scale clinical research to better investigate if the association between RBC folate and CHF is a cause-effect relationship, what are the underlying pathophysiological basis, as well as to identify optimal dietary folate equivalent (DFE) and RBC folate concentration intervals.

## 1. Introduction

Congestive heart failure (CHF), a type of cardiovascular disease (CVD), is a complex clinical syndrome with symptoms and signs that result from any structural or functional impairment of ventricular filling or the ejection of blood [1]. Presently, approximately 6 million individuals aged 20 and above in the United States have CHF [2]. Folate is an essential nutrient required for complex biochemical reactions such as nucleotide synthesis and methyl group transfer [3, 4]. Hyperhomocysteine, which is mainly caused by folate deficiency [5], has been widely demonstrated to increase the risk of CHF [5, 6, 7, 8, 9, 10]. The existing body of evidence substantiates that folate intake may reduce plasma homocysteine (Hcy) concentrations [11, 12]. Furthermore, research has revealed a marked contrast in folate consumption between CHF patients and healthy subjects [13].

All dietary folate functions biologically through absorption and conversion to active forms of folate in the body. Red blood cell (RBC) folate is a reliable biomarker of long-lasting folate status and is the endorsed gold standard by the World Health Organization [14]. Notably, a study found that compared with the lowest quintile of RBC folate, the highest quintile was associated with higher CVD mortality [15]. However, prior studies have not yet quantified the relationship between this biomarker and CHF risk. Therefore, it remains unclear whether and to what extent RBC folate concentration is associated with CHF risk. To address this question, our study employed data from a cross-sectional analysis of the National Health and Nutrition Examination Survey (NHANES) to elucidate the intricate association between RBC folate levels and risk of CHF.

## 2. Materials and Methods

### 2.1 Study Population

We used data from the 2011–2020 NHANES, a population-based national cross-sectional survey carried out by the United States Centers for Disease Control and Prevention (CDC). The NHANES consists of examination, interview, and laboratory data. It has a complicated design featuring stratification, multiple stages, and clustered sampling of probability using a non-institutional and nationally representative American civilian population survey.

**Fig. 1.**
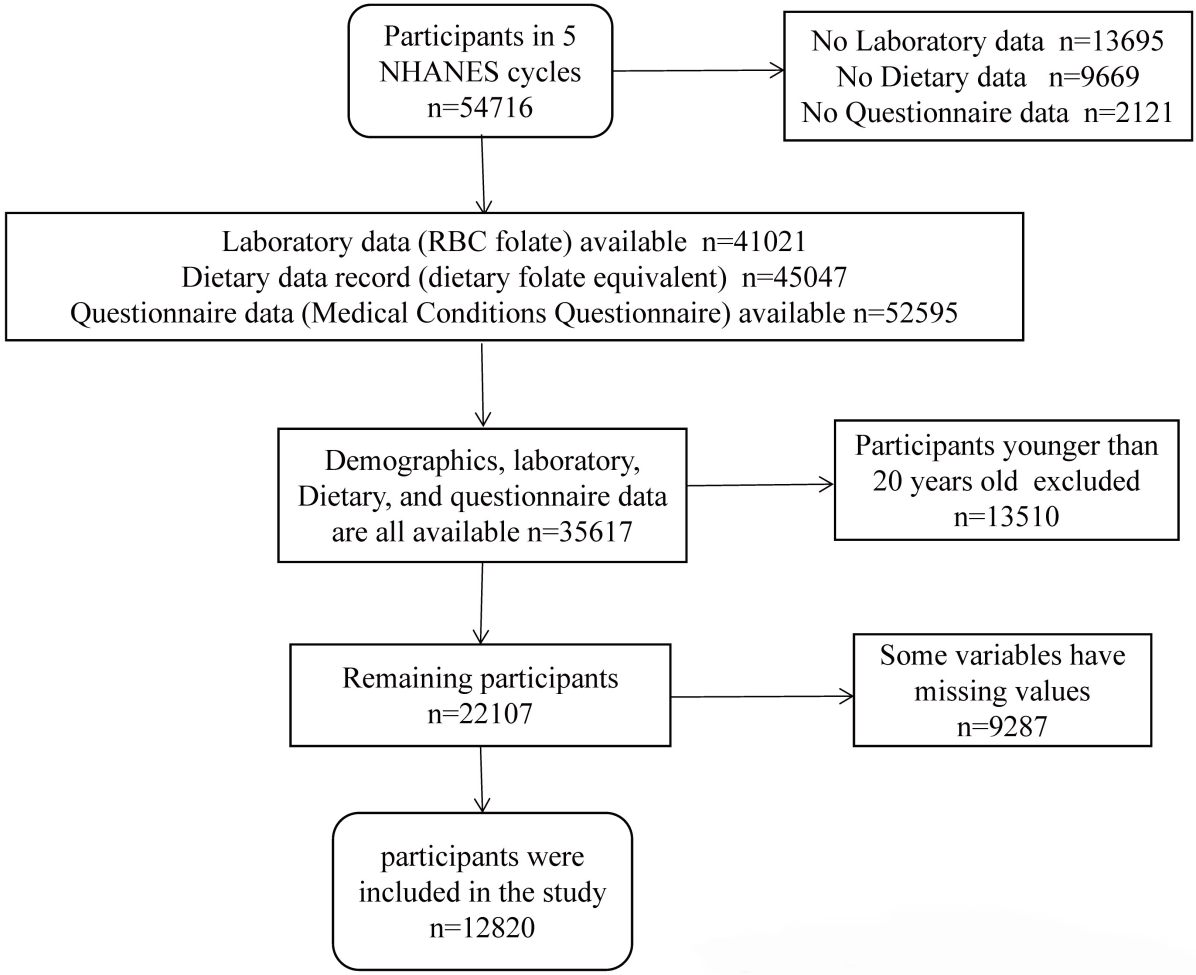
**Flowchart of the participants**. NHANES, the National Health and Nutrition Examination Survey; RBC, red blood cell.

A total of 54,716 individuals participated in the NHANES from 2011 to 2020. A total of 41,021 participants provided laboratory data on RBC folate, 45,047 individuals provided dietary folate data, and 52,595 individuals completed the Medical Conditions Questionnaire (MCQ). Demographic, laboratory, questionnaire, and dietary data were available for 35,617 individuals after integration. Individuals under the age of 20 years (n = 13,510) or with missing data (n = 9287) were excluded from the study. Finally, 12,820 individuals were included in the study. The data screening process is shown in Fig. 1. The specific statistics for the missing values are shown in **Supplementary Fig. 1**.

### 2.2 Assessment of CHF

Participants who answered yes to the MCQ160b questionnaire were considered to have CHF. The use of self-reported CHF as a method to identify a nationally representative cohort of patients with CHF can be considered a reasonable approach [16]. This is supported by the fact that self-reported heart failure (HF) data from the NHANES are included in the American Heart Association’s annual report on CVD and stroke [16]. The specificity of self-reported HF was previously demonstrated to be greater than 99% [17]. Furthermore, self-reported HF has also been used in some studies on HF using the NHANES database [18, 19].

### 2.3 Assessment of RBC and Dietary Folate Data

The blood samples were handled, frozen at –20 °C, and delivered to the National Center for Environmental Health for examination. A detailed summary of the laboratory methodology can be found on the NHANES website. Because RBC folate is a better indicator of long-term folate status than serum folate [20], we used RBC folate levels in this study. Microbiological assays have been used to measure RBC folate concentrations. Participants were divided into three groups based on RBC folate: lowest tertile group (T1), middle tertile group (T2), and highest tertile group (T3).

The assessment of dietary folate was conducted through the examination of 24-h recall records. The NHANES carried out two evaluations. The initial data collection occurred at a mobile examination center, whereas the subsequent assessment was conducted through telephone interviews after 3–10 days. Research using biomarkers has shown that the 24-h food recalls dietary assessment method has less bias in the assessment of dietary intake than the food frequency questionnaire [21]. Sources of folate intake included naturally occurring folate in food and folic acid, which is the synthetic form of folate. The two bioavailabilities are different [22]. Therefore, the total daily intake of folate and folic acid needs to be translated to dietary folate equivalents (DFEs) to integrate the two sources and account for the higher absorption of folic acid. The equations for calculating the DFE are as follows: Total daily DFE intake (mcg) = [average of food folate reported in the two days of the recall] (µg) + [average of folic acid from fortification and dietary supplements reported in the 2 days of dietary recall] × 1.7 (µg) [22]. Considering that the current recommended daily allowance (RDA) of DFE intake is 400 mcg and the tolerable upper intake level (UL) is 1000 mcg, we used DFE data to divide the population into three groups according to RDA and UL standards (insufficient, <400 mcg; standard, 400–1000 mcg; excess, >1000 mcg).

### 2.4 Assessment of Covariates

Some variables of sociodemographic characteristics and health-related status were included in the statistical analysis models to adjust for the potential influence of confounding variables. Variables included age (<65, ≥65), sex (male and female), ethnicity (Mexican American, White, Black, others), educational level (high school or below, college degree and above), marital status (married, others), annual household income (<$35,000, $35,000–$74,999, ≥$75,000), health insurance (yes, no), body mass index (BMI) (<25 ${\mathrm{kg/m}}^{2}$, ≥25 ${\mathrm{kg/m}}^{2}$), stroke (yes, no), diabetes mellitus (DM) (DM, impaired fasting glucose, impaired glucose tolerance, no), hypertension (yes, no), hyperlipidemia (yes, no), alcohol consumption (never, former, mild, moderate, heavy), and smoking status (yes, no). These data were obtained from NHANES-related questionnaires or demographic data. All diagnostic methods and grading information are available on the official NHANES website (https://www.cdc.gov/nchs/nhanes/). Folate deficiency is defined by a cut-off value <317 nmol/L for RBC folate concentration [23].

### 2.5 Statistical Analyses

All analyses utilized weighted samples and took into account the clustering and stratification of designs to obtain estimates that were applicable to the United States [24]. To provide estimates for the entire 10-year study period, we created a sample of weight variables over 10 years by taking one-fifth of each participant’s weight over 2 years.

For the baseline survey, selected characteristics were presented as mean and standard deviation (continuous variables) or frequency distribution (categorical variables). Analysis of variance for continuous variables and chi-squared tests for categorical variables were applied to test the significance levels of the differences. Univariate and multivariate logistic regression analyses were used to examine the association between RBC folate and the risk of CHF, and odds ratios (ORs) and 95% confidence intervals (CIs) were calculated. The group of individuals whose RBC folate was identified in the lowest tertile was used as the reference. We created models with no adjusted covariates (Model 1); models adjusted solely for sex, age, and ethnicity (Model 2); and models further adjusted for DFE, sex, age, ethnicity, education level, annual household income, marital status, health insurance, smoking status, alcohol consumption, BMI, DM, stroke, hypertension, and hyperlipidemia (Model 3). As many as 3809 patients were excluded from the study simply because of a missing covariate, annual household income. So we removed the covariate of household income for the sake of a larger sample size, retained these participants, and reran the three model analyses mentioned above. We also analyzed the relationship between folate deficiency and CHF after adjusting for the multiple covariates mentioned in Model 3. Moreover, we stratified the analysis by age, sex, ethnicity, BMI, and smoking status. Finally, we constructed a complex model adjusted previously mentioned covariates to predict the dose-response relationship between RBC folate and CHF by using the restrictive cubic spline (RCS) method. We chose the knot when the Akaike information criterion value was the minimum.

R Statistical Software (version 4.3.1, R Foundation for Statistical Computing, Vienna, Austria) was used for all analyses. *p* < 0.05 (two-tailed) was considered statistically significant.

## 3. Results

### 3.1 Baseline Characteristics

A total of 9287 participants were excluded due to the presence of missing values, and these participants were younger and had a greater proportion of females and blacks than those included in the study. The specific missing values are shown in **Supplementary Fig. 1**. The 12,820 NHANES participants represented 143.06 million non-institutionalized residents of the United States. The average age of the participants was 49.34 ± 17.45 years, of whom 52.8% were women. A total of 408 patients with CHF accounted for 3.18%. In the different tertile groups of RBC folate, age, DFE, BMI, sex, ethnicity, prevalence rate, folate deficiency, annual household income, education level, marital status, health insurance, DM, hyperlipidemia, hypertension, stroke, smoking status, and alcohol consumption were significantly different. Compared with the lowest tertile group of RBC folate, participants in the highest tertile group were older on average, had more daily DFE, were more likely to be female, and had a higher prevalence of CHF. The characteristics of the study sample are presented in Table 1.

**Table 1.** **Baseline characteristics of NHANES participants**.

Variables			Stratified by RBC folate (nmol/L)			*p-*value
			T1	T2	T3	
N		12,820	4257	4206	4357	
Age (years) (mean [SD])		49.3 (17.5)	44.9 (16.7)	47.6 (16.8)	55.4 (17.1)	<0.001
DFE (mcg/d) (mean [SD])		512.3 (310.0)	465.3 (286.9)	523.5 (310.9)	547.4 (325.1)	<0.001
BMI (${\mathrm{kg/m}}^{2}$) (mean [SD])		29.5 (7.2)	29.1 (7.4)	29.5 (7.1)	29.9 (7.0)	<0.001
Sex (%)						
	Female	6765 (52.8)	2192 (51.5)	2156 (51.3)	2417 (55.5)	<0.001
	Male	6055 (47.2)	2065 (48.5)	2050 (48.7)	1940 (44.5)	
Ethnicity (%)						
	Mexican	1650 (12.9)	544 (12.8)	647 (15.4)	459 (10.5)	<0.001
	Black	2828 (22.1)	1408 (33.1)	808 (19.2)	612 (14.0)	
	White	5296 (41.3)	1256 (29.5)	1658 (39.4)	2382 (54.7)	
	Other	3046 (23.8)	1049 (24.6)	1093 (26.0)	904 (20.7)	
CHF (%)						
	No	12,412 (96.8)	4167 (97.9)	4090 (97.2)	4155 (95.4)	<0.001
	Yes	408 (3.2)	90 (2.1)	116 (2.8)	202 (4.6)	
Folate deficiency (%)						
	No	12,772 (99.6)	4209 (98.9)	4206 (100.0)	4357 (100.0)	<0.001
	Yes	48 (0.4)	48 (1.1)	0 (0.0)	0 (0.0)	
Income (%)						
	$35,000–$74,999	956 (7.5)	322 (7.6)	300 (7.1)	334 (7.7)	<0.001
	<$35,000	10,023 (78.2)	3404 (80.0)	3281 (78.0)	3338 (76.6)	
	≥$75,000	1841 (14.4)	531 (12.5)	625 (14.9)	685 (15.7)	
Education (%)						
	≥College degree	3761 (29.3)	1204 (28.3)	1209 (28.7)	1348 (30.9)	0.015
	≤High school	9059 (70.7)	3053 (71.7)	2997 (71.3)	3009 (69.1)	
Marital status (%)						
	Married	6561 (51.2)	1859 (43.7)	2203 (52.4)	2499 (57.4)	<0.001
	Others	6259 (48.8)	2398 (56.3)	2003 (47.6)	1858 (42.6)	
Health insurance (%)						
	No	2444 (19.1)	1038 (24.4)	859 (20.4)	547 (12.6)	<0.001
	Yes	10,376 (80.9)	3219 (75.6)	3347 (79.6)	3810 (87.4)	
DM (%)						
	DM	2449 (19.1)	631 (14.8)	745 (17.7)	1073 (24.6)	<0.001
	IFG	596 (4.6)	194 (4.6)	202 (4.8)	200 (4.6)	
	IGT	470 (3.7)	146 (3.4)	127 (3.0)	197 (4.5)	
	No	9305 (72.6)	3286 (77.2)	3132 (74.5)	2887 (66.3)	
Hyperlipidemia (%)						
	No	3857 (30.1)	1464 (34.4)	1333 (31.7)	1060 (24.3)	<0.001
	Yes	8963 (69.9)	2793 (65.6)	2873 (68.3)	3297 (75.7)	
Hypertension (%)						
	No	7288 (56.8)	2675 (62.8)	2570 (61.1)	2043 (46.9)	<0.001
	Yes	5532 (43.2)	1582 (37.2)	1636 (38.9)	2314 (53.1)	
Stroke (%)						
	No	12,353 (96.4)	4131 (97.0)	4076 (96.9)	4146 (95.2)	<0.001
	Yes	467 (3.6)	126 (3.0)	130 (3.1)	211 (4.8)	
Smoker (%)						
	No	10,430 (81.4)	3174 (74.6)	3439 (81.8)	3817 (87.6)	<0.001
	Yes	2390 (18.6)	1083 (25.4)	767 (18.2)	540 (12.4)	
Alcohol consumption (%)						
	Former	1878 (14.6)	551 (12.9)	569 (13.5)	758 (17.4)	<0.001
	Heavy	2419 (18.9)	926 (21.8)	865 (20.6)	628 (14.4)	
	Mild	4596 (35.9)	1452 (34.1)	1489 (35.4)	1655 (38.0)	
	Moderate	2111 (16.5)	746 (17.5)	726 (17.3)	639 (14.7)	
	Never	1816 (14.2)	582 (13.7)	557 (13.2)	677 (15.5)	

CHF, congestive heart failure; BMI, body mass index; DFE, dietary folate equivalent; DM, diabetes mellitus; IFG, impaired fasting glucose; IGT, impaired glucose tolerance; SD, standard deviation; NHANES, the National Health and Nutrition Examination Survey; RBC, red blood cell; N, number; T1, lowest tertile group; T2, middle tertile group; T3, highest tertile group.

### 3.2 Association of RBC Folate Concentration and DFE with CHF

In the three different tertile groups, there were 90, 116, and 202 cases of CHF, respectively. Overall, the prevalence of CHF increased progressively from the low to high folate concentration group (T1: 2.11%, T2: 2.76%, T3: 4.64%). The unadjusted model showed that the highest tertile group of RBC folate concentration was significantly associated with a higher risk of CHF compared to the lowest tertile group of RBC folate levels (OR = 3.09; 95% CI, 2.14–4.46). The harmful association remained after adjusting for age, sex, and ethnicity (OR = 1.75; 95% CI, 1.16–2.63). Similar trends were seen in the multivariate-adjusted analysis (OR = 1.98; 95% CI: 1.27–3.09) (Table 2). Compared with the lowest tertile group of RBC folate concentration, the prevalence of CHF in the highest tertile group increased by 98%. In addition, we found that inadequate DFE (<400 mcg/d) intake was also associated with an increased risk of CHF compared with standard DFE (OR = 1.63; 95% CI: 1.22–2.17). However, as DFE continued to increase and above UL (1000 mcg), the association with CHF was no longer significant. The specific results of the multivariate adjustment model can be found in **Supplementary Fig. 2**.

**Table 2.** **Association between RBC folate (nmol/L) and CHF, stratified by sex**.

		Events/PR (n/%)	Model 1 OR (95% CI)	Model 2 OR (95% CI)	Model 3 OR (95% CI)
Total					
	T1	90/2.11%	Ref	Ref	Ref
	T2	116/2.76%	1.43 (1.01, 2.04)	1.31 (0.92, 1.86)	1.51 (1.00, 2.27)
	T3	202/4.64%	3.09 (2.14, 4.46)	1.75 (1.16, 2.63)	1.98 (1.27, 3.09)
*p* for trend			*p* < 0.001	*p* = 0.01	*p* = 0.005
	Male				
	T1	43/2.11%	Ref	Ref	Ref
	T2	66/3.26%	1.44 (0.80, 2.59)	1.38 (0.75, 2.54)	1.52 (0.76, 3.06)
	T3	105/5.47%	3.26 (1.86, 5.70)	1.72 (0.93, 3.17)	1.85 (0.93, 3.71)
*p* for trend			*p* < 0.001	*p* = 0.09	*p* = 0.09
	Female				
	T1	46/2.10%	Ref	Ref	Ref
	T2	48/2.23%	1.42 (0.86, 2.35)	1.26 (0.76, 2.10)	1.58 (0.87, 2.86)
	T3	96/3.97%	2.95 (1.94, 4.51)	1.79 (1.09, 2.96)	2.11 (1.26, 3.54)
*p* for trend			*p* < 0.001	*p* = 0.02	*p* = 0.005

PR, prevalence rate; OR, odds ratio; CI, confidence interval; CHF, congestive heart failure; BMI, body mass index; CHF, congestive heart failure; DFE, dietary folate equivalent; DM, diabetes mellitus; RBC, red blood cell; T1, lowest tertile group; T2, middle tertile group; T3, highest tertile group. Model 1: Unadjusted model. Model 2: Sex, age, and ethnicity adjusted model. Model 3: Multivariate-adjusted model including DFE, sex, age, ethnicity, education levels, annual household incomes, marital status, health insurance, smoking status, alcohol consumption, BMI, DM, stroke and hypertension, and hyperlipidemia.

The association remained unchanged in women. Compared to the lowest tertile group of RBC folate levels, the highest tertile group of RBC folate concentration was significantly associated with a higher risk of CHF, with an OR (95% CI) of 2.95 (1.94, 4.51) for unadjusted, 1.79 (1.09, 2.96) for age, sex, and ethnicity adjusted, and 2.11 (1.26, 3.54) for the multivariate-adjusted model. However, this relationship did not exist in men. Only the unadjusted model showed such an association with OR (95% CI) of 3.26 (1.86, 5.70); the relationship between RBC folate and CHF was no longer significant after adjustment. The same relationship existed after increasing the sample size by retaining participants who were excluded due to missing annual household income (**Supplementary Table 1**).

Only 48 patients had folate deficiency, with a weighted prevalence of 3.7‰. We found that folate deficiency could increase the risk of CHF after adjusting for DFE, sex, age, ethnicity, education level, annual household income, marital status, health insurance, smoking status, alcohol consumption, BMI, DM, stroke and hypertension, and hyperlipidemia (OR = 4.92; 95% CI, 1.11–21.8, *p* = 0.04).

**Fig. 2.**
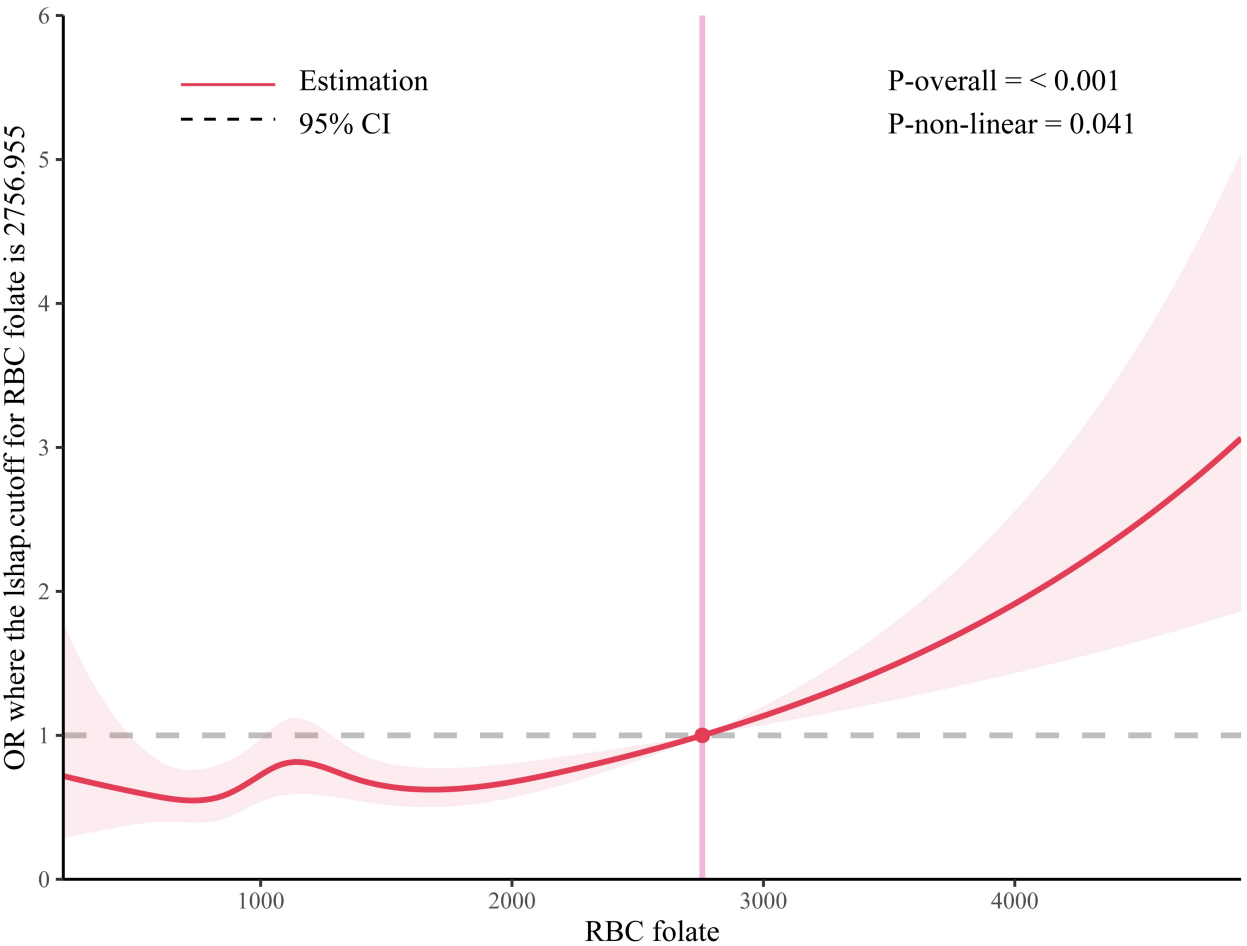
**The RCS analysis of red blood cell (RBC) folate concentrations and risk of congestive heart failure (CHF)**. RCS, restrictive cubic spline; BMI, body mass index; CI, confidence interval. Models adjusted for dietary folate equivalent (DFE), sex, age, ethnicity, education levels, annual household incomes, marital status, health insurance, smoking status, alcohol consumption, BMI, diabetes mellitus (DM), stroke and hypertension, and hyperlipidemia. We chose the five knots when the Akaike information criterion (AIC) value was the minimum. The odds ratio (OR) was >1.0 when the predicted RBC folate level was >2757 nmol/L (*p*-value for non-linearity = 0.001).

In addition, we also performed quantitative analyses. We used a complicated RCS model and visualized the predicted relationship between RBC folate and risk of CHF. The OR was >1.0 when the predicted RBC folate exceeded 2757 nmol/L (Fig. 2). The risk of CHF was low and relatively stable until the level of predicted RBC folate was 2757 nmol/L but began to increase rapidly thereafter (*p* = 0.001), which suggests that excessive RBC folate can increase the risk of CHF.

### 3.3 Association of RBC Folate Concentration with CHF Stratified by Participants’ Features 

**Fig. 3.**
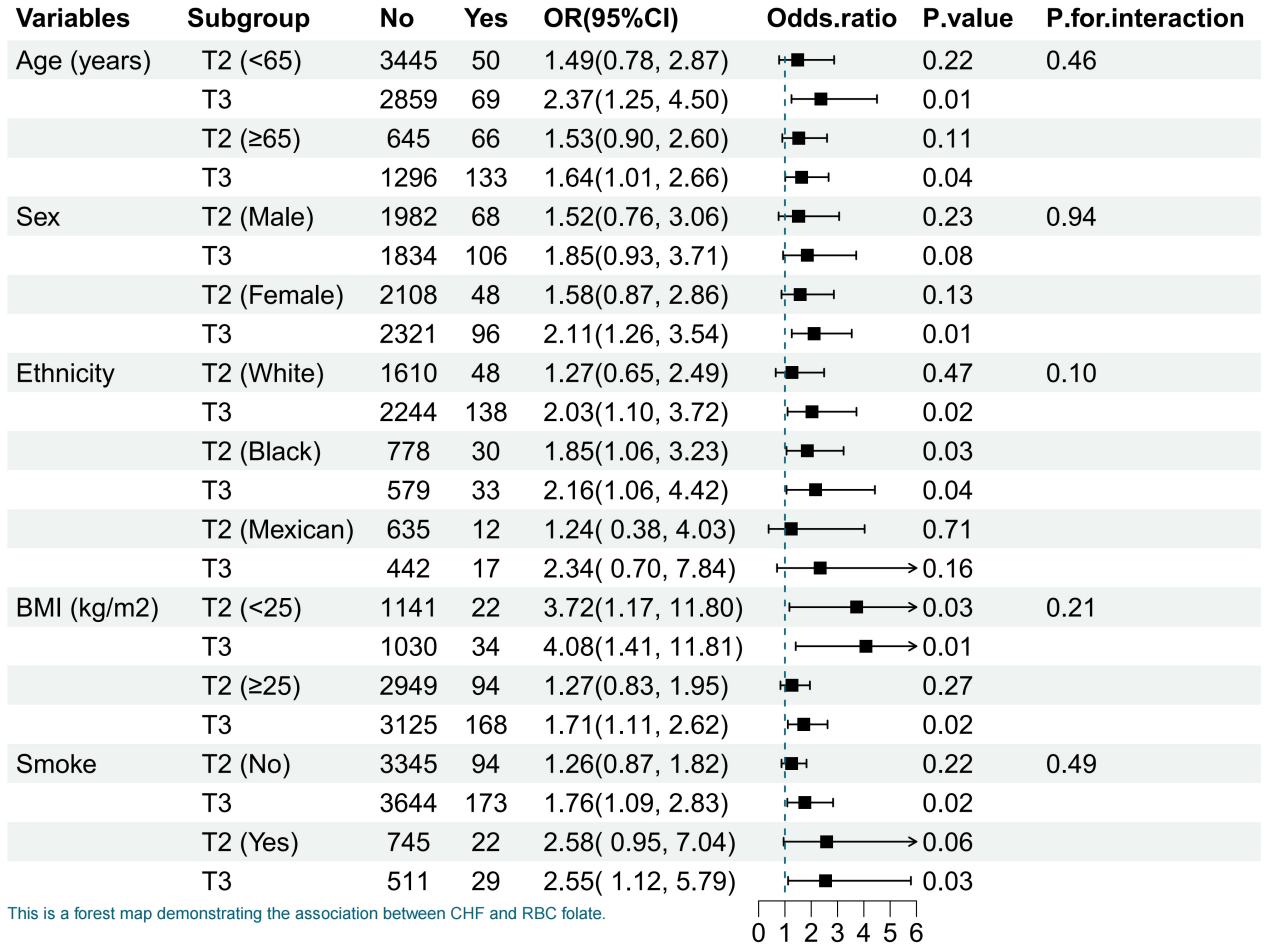
**Association of RBC folate tertile with CHF stratified by participant’s characteristics**. OR, odds ratio; CI, confidence interval; BMI, body mass index; CHF, congestive heart failure; RBC, red blood cell; DFE, dietary folate equivalent; DM, diabetes mellitus; T2, middle tertile group; T3, highest tertile group. The multivariate-adjusted model included DFE, sex, age, ethnicity, education levels, annual household incomes, marital status, health insurance, smoking status, alcohol consumption, BMI, DM, stroke and hypertension, and hyperlipidemia.

We stratified the analysis by age, sex, ethnicity, BMI, and smoking status. The results were broadly consistent with our previous finding that the highest tertile group, compared with the lowest tertile group, may increase the risk of CHF (Fig. 3). There was no interaction across all subgroups. However, this relationship was only significant in females (OR = 2.11; 95% CI: 1.26–3.54) and not in males (OR = 1.85; 95% CI: 0.93–3.71). In addition to differences in sex subgroups, there were also differences in ethnic subgroups. In the multivariate-adjusted model, the association was present only in White (OR = 2.03; 95% CI: 1.10–3.72) and Black (OR = 2.16; 95% CI: 1.06–4.42) participants.

## 4. Discussion

Based on data from a nationally representative United States survey across 10 years (2011–2020), we investigated the relationship between RBC folate and the prevalence of CHF. To the best of our knowledge, this is the first study to extensively explore this relationship using a world-class dataset. Notably, our findings suggest the dual nature of RBC folate’s impact on CHF risk: either excessive levels (the highest tertile) or deficiency in RBC folate might increase the risk of CHF.

Multivariate-adjusted logistic regression analysis revealed several factors associated with an increased risk of CHF, including high RBC folate concentrations (the highest tertile or >2757 nmol/L), folate deficiency (<317 nmol/L), insufficient dietary folate (mcg <40 µg), older age, male sex, smoking status, DM, hyperlipidemia, hypertension, history of stroke, and annual household income under $35,000. While further discussion is warranted for each identified factor, this research focused specifically on delineating the relationship between RBC folate concentrations and CHF risk.

A novel finding of this study is that the prevalence of CHF was higher in the highest tertile group compared to the lowest tertile group of RBC folate concentration. Our findings remained consistent and robust across both univariate and multivariate logistic regression models, as well as subgroup analyses, demonstrating an increased risk of CHF associated with the highest tertile of RBC folate concentrations. However, it is important to note that this trend was not consistently observed within certain subgroups in the Mexican-American or male populations. Interestingly, there was no interaction between all subgroups. Therefore, we determined whether the threshold at which elevated RBC folate concentrations would increase the risk of CHF in male and Mexican-American populations would not be consistent with other subgroups. To test this hypothesis, we divided the male and Mexican-American populations into four groups by the quartiles of RBC folate and then reran the model. We found that the highest quartile of RBC folate increased the risk of CHF compared with the lowest quartile (OR = 2.26; 95% CI: 1.12–4.56) in male populations, and a similar trend was also found in Mexican-Americans (OR = 3.49; 95% CI: 1.00–12.14). Thus, the conclusion that high levels of RBC folate may increase the risk of CHF is consistent and reliable. The results of the quantitative analyses of RCS were also consistent. The risk of CHF was low and relatively stable up to a predicted RBC folate level of 2757 nmol/L, but then began to increase rapidly, suggesting that excessive RBC folate may increase the risk of CHF. Although no studies have found that high levels of RBC folate may increase the risk of CHF, one study showed that high levels of RBC folate may increase CVD mortality [15]. A study from NHANES spanning six cycles from 2003 to 2014 with a total of 14,234 participants with high-risk factors for CVD found that compared with the lowest quintile of RBC folate, the highest quintile was associated with higher CVD mortality (hazard ratio: 1.40, 95% CI: 1.02–1.93; *p* = 0.030) [15].

Research suggests that high RBC folate concentrations may increase the risk of CHF possibly due to high folate levels impairing normal folate physiological function [25]. Excessive folate will accumulate in the circulation if it is not utilized, thus reducing the formation of thymidylate and leading to the inhibition of aberrant DNA methylation [26] and DNA synthesis [25]. Another possible reason is that high concentrations of RBC folate may inhibit folate-dependent enzymes for which RBC folate is a substrate, thereby affecting normal biochemical reactions [27]. Furthermore, excessive RBC folate can induce the cytotoxicity of natural killer (NK) cells [28]. The activation of NK cells can lead to the secretion of proinflammatory cytokines [29], possibly resulting in increased risk of CVD and all-cause mortality [30, 31, 32]. These factors may represent mechanisms underlying the harmful effects of a folate overdose, although this still needs to be verified.

It has been almost 24 years since mandatory folic acid fortification was introduced in the United States. The prevalence of folate deficiency persisted at <1% over the entire 25-year period [23]. Our study included only 48 participants with folate deficiency. After performing multivariate logistic regression analysis with CHF, the resulting CI was wide. Thus, our study did not present sufficient evidence that folate deficiency can increase the risk of CHF. Nevertheless, previous studies suggest that folate deficiency may increase the risk of CHF [5, 33]. And folate is a free radical scavenger that acts as an antioxidant, protecting the organism from damage caused by the accumulation of free radicals [34]. Its antioxidant role is very important in CVD [35]. In addition to oxidative stress, a deficiency of one or more B vitamins (including folate) may impair ATP production. ATP depletion causes the weakening of cardiac muscle, which eventually leads to HF [36]. A prospective cohort study found that elevated total Hcy concentration independently predicted the risk of occurrence of CHF in adults [9]. Hyperhomocysteinemia can damage the endothelium and blood vessel wall, having a negative impact on the mechanisms underlying CHF, such as oxidative stress and inflammation [37]. Some animal experiments have shown that hyperhomocysteinemia might cause myocardium fibrosis and diastolic dysfunction in rats [38, 39]. In addition to high Hcy, some studies have shown that uric acid (UA) is positively associated with an increased risk of incident CHF [40, 41]. However, folate and its derivatives may reduce UA by inactivating xanthine oxidoreductase, the enzyme responsible for the oxidation of hypoxanthine to xanthine and xanthine to UA [42]. Thus, folate may also reduce the risk of CHF by lowering UA. It is not difficult to deduce that folate deficiency may therefore increase the risk of CHF. It is worth mentioning that the low RBC folate concentration in our study was just relative to the total population, which is the concentration after folic acid fortification, and not equivalent to folate deficiency. Therefore, the low RBC folate concentration in our study may not increase the risk of CHF. This may be the reason why the risk of CHF is not greater at the lower levels of RBC folate in the RCS curve.

This study showed that insufficient DFE (<400 mcg) intake also increased the risk of CHF, consistent with previous prospective studies that reported the inverse association of increasing dietary folate intake with lower CVD mortality [15, 43]. Some studies have shown that increased DFE intake also reduces the risk of hyperuricemia [44] and hyperhomocysteinemia [45], which may ultimately reduce CHF risk. However, the cardioprotective effect did not increase proportionally as DFE intake was above the UL. Notably, the correlation between RBC folate concentration and DFE was found to be weak (Spearman’s r = 0.14, *p* < 0.001), as RBC folate is influenced by numerous factors such as fortified products or supplements containing folic acid [46], folate requirements, absorption [46], and polymorphisms in folate metabolizing enzymes [47, 48], sex [46], age [49], and smoking [50]. Furthermore, in a study conducted by the NHANES, even though DFE intake remained the same, there was a notable increase in RBC folate concentrations as individuals aged [51]. Older subjects are more likely to develop CHF. In our study, 61.5% of CHF patients were older than 65 years of age, and these CHF patients still had higher RBC folate concentrations even with insufficient folate intake. Therefore, we should focus on DFE in the elderly to avoid excessive intake and an increase in CHF risk.

Our analyses found that folate deficiency exacerbates the risk of CHF. This illustrates the protectiveness and effectiveness of the folic acid fortification programs. In consideration of this, we believe that folic acid fortification should continue to be vigorously implemented. Following the RDA, we found that insufficient DFE is associated with an increased risk of CHF, thus requiring clinicians, health managers, and nurses to promote people to achieve the RDA. However, considering the two sides of RBC folate for CHF, to avoid excessive folate intake and high RBC folate concentration, there is a critical need for large-scale clinical research to identify safe DFE and RBC folate concentration intervals. In light of the prevalence of CHF and the associated morbidity and mortality, nutritional strategies to promote appropriate amounts are important areas for further research [52]. Furthermore, DFE and RBC folate levels should also be considered in the overall management of patients with CHF.

This study had several limitations. Firstly, our findings can be generalized to the United States population only. Secondly, the present study had a cross-sectional design. Thus, our findings are limited to the potential association but not causation between CHF and RBC folate, and the underlying mechanism remains elusive. Thirdly, excluding about 50% of participants due to missing data is a limitation which could potentially impact the final quantitative results. Finally, there is a restriction in particular due to the sensitivity of self-reported CHF. Patients did not report having CHF until they went to the hospital and were diagnosed with CHF by a doctor, and patients with less severe CHF who were not hospitalized and who did not have a definitive CHF diagnosis may have been unaware they have CHF and may not have reported CHF.

## 5. Conclusions

The risk of CHF may be increased either by high RBC folate concentrations (highest tertile of RBC folate or >2637 nmol/L) or by folate deficiency. Considering the two sides of the association between RBC folate and CHF, there is a need for large-scale clinical research to better investigate if the association between RBC folate and CHF is a cause-effect relationship, what are the underlying pathophysiological basis, as well as to identify optimal DFE and RBC folate concentration intervals.

## Data Availability

All data is open source and all data is available from the website at the following address: https://wwwn.cdc.gov/Nchs/Nhanes/.

## Abbreviations

CHF, congestive heart failure; RBC, red blood cell; DFE, dietary folate equivalent; NHANES, National Health and Nutrition Examination Survey; RCS, restrictive cubic spline; OR, odds ratio; CI, confidence interval; ATP, adenosine triphosphate; Hcy, homocysteine; MCQ, Medical Conditions Questionnaire; RDA, recommended daily allowance; UL, tolerable upper intake level; CDC, Centers for Disease Control and Prevention; BMI, body mass index; DM, diabetes mellitus.

## Supplementary Material

Supplementary material associated with this article can be found, in the online version, at https://doi.org/10.31083/j.rcm2502039.
