# Supplementary material for: Folate in the United States Population and its Association with Congestive Heart Failure
Source: Rev Cardiovasc Med. 2024 Jan 29;25(2):39. doi: 10.31083/j.rcm2502039 (PMC11263171; doi:10.31083/j.rcm2502039)
Supplement: Supplementary file 1 [file 2153-8174-25-2-039-s1.docx]

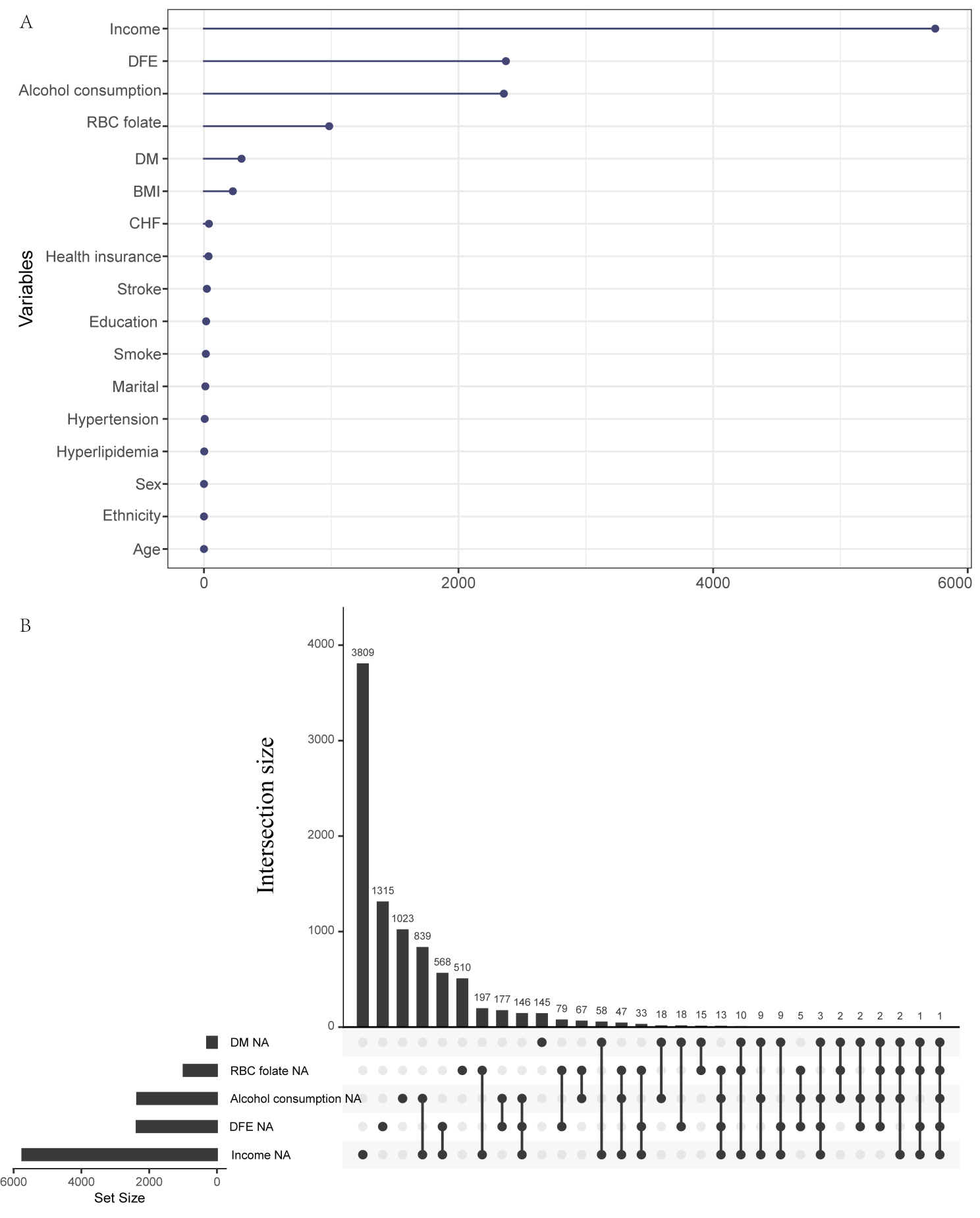


**Supplementary Fig. 1. Missing value specifics. (**Figure A) Number of missing values for each different variable. (Figure B0 Overlap of the five variables with the highest number of missing values. For example, the first column from the left indicates that 3809 participants were excluded because only one variable, annual household income, was missing, and the fourth column indicates that 839 patients were excluded because both annual household income and alcohol consumption variables were missing.


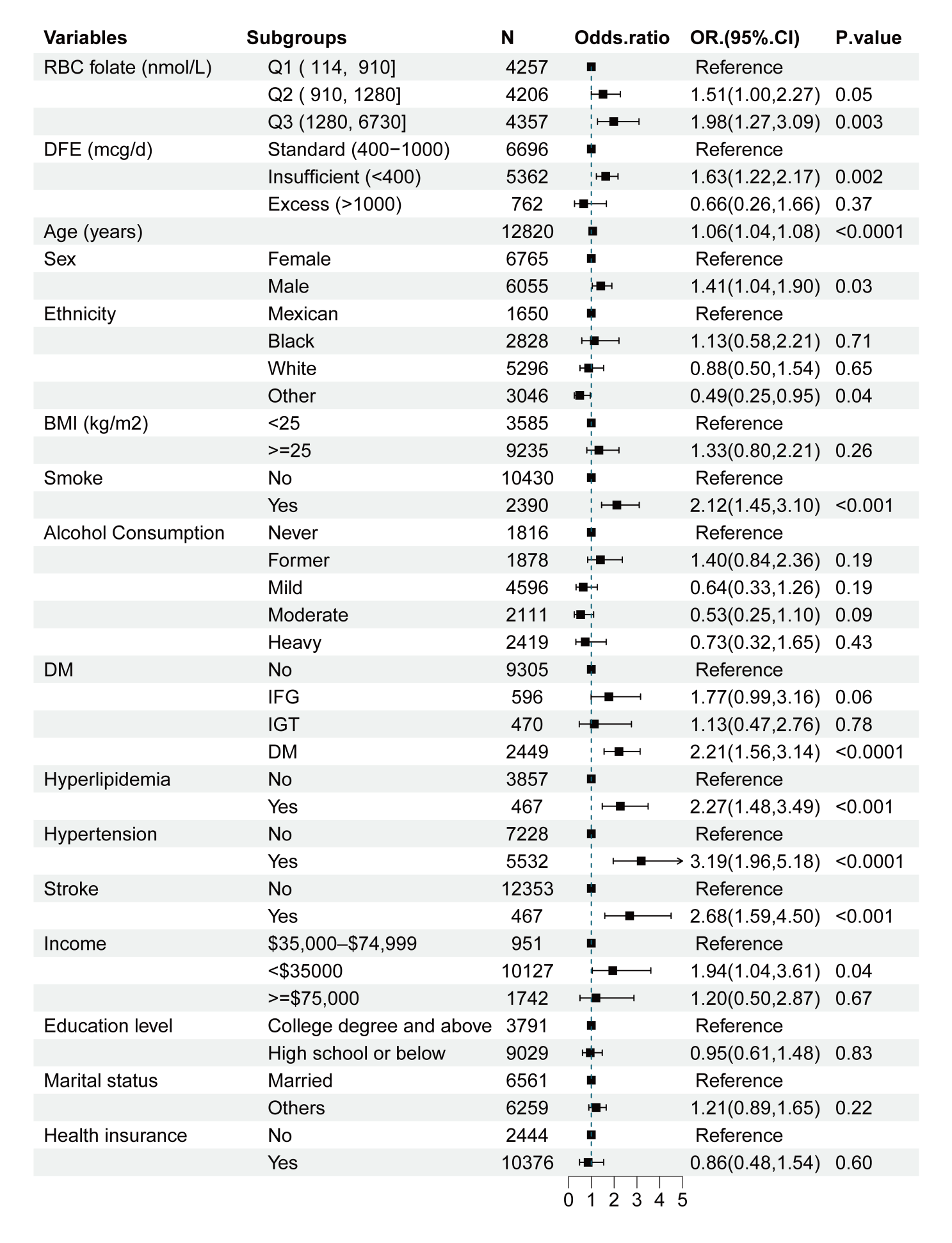


**Supplementary Fig. 2. Association of RBC folate tertile with CHF.** OR: odds ratio; Cl: confidence interval; BMI: body mass index. The multivariate-adjusted model included DFE, sex, age, ethnicity, education levels, annual household incomes, marital status, health insurance, smoking status, alcohol consumption, BMI, DM, stroke and hypertension, and hyperlipidemia.

Supplementary Table 1. Association between RBC folate (nmol/L) and CHF, stratified by sex. (Increase of 3809 participants).

|  | Events/PR (n/%) | Model 1 OR (95% CI) | Model 2 OR (95% CI) | Model 3 OR (95% CI) |
| --- | --- | --- | --- | --- |
| Total |  |  |  |  |
| T1 | 118/2.14% | Ref | Ref | Ref |
| T2 | 142/2.59% | 1.37(0.99,1.90) | 1.26(0.90,1.75) | 1.45(0.99,2.12) |
| T3 | 241/4.33% | 2.79(2.02,3.85) | 1.59(1.12,2.27) | 1.75(1.20,2.56) |
| P for trend |  | P< 0.001 | P=0.02 | P=0.007 |
| Male |  |  |  |  |
| T1 | 59/2.30% | Ref | Ref | Ref |
| T2 | 85/3.27% | 1.46(0.87,2.44) | 1.39(0.81,2.39) | 1.58(0.89,2.79) |
| T3 | 126/5.23% | 2.87(1.78,4.63) | 1.52(0.89,2.58) | 1.57(0.89,2.79) |
| P for trend |  | P< 0.001 | P=0.16 | P=0.18 |
| Female |  |  |  |  |
| T1 | 59/2.00% | Ref | Ref | Ref |
| T2 | 57/1.97% | 1.25(0.80,1.96) | 1.13(0.71,1.79) | 1.32(0.75, 2.32) |
| T3 | 115/3.64 | 2.73(1.89,3.92) | 1.68(1.09,2.57) | 1.86(1.19, 2.90) |
| P for trend |  | P< 0.001 | P=0.01 | P=0.003 |

Increase of 3809 participants who were removed solely because of the absence of a variable on annual household income

PR: prevalence rate; OR: odds ratio; Cl: confidence interval

Model 1: Unadjusted model.

Model 2: Sex, age, and ethnicity adjusted model.

Model 3: Multivariate-adjusted model included DFE, sex, age, ethnicity, education levels, marital status, health insurance, smoking status, alcohol consumption, BMI, DM, stroke and hypertension, and hyperlipidemia.
